# Early recognition of the 2009 pandemic influenza A (H1N1) pneumonia by chest ultrasound

**DOI:** 10.1186/cc11201

**Published:** 2012-02-17

**Authors:** Americo Testa, Gino Soldati, Roberto Copetti, Rosangela Giannuzzi, Grazia Portale, Nicolò Gentiloni-Silveri

**Affiliations:** 1 Department of Emergency Medicine, A. Gemelli University Hospital, Rome, Italy; 2 Operative Unit of Emergency Medicine, Castelnuovo Garfagnana Hospital, Lucca, Italy; 3 Department of Emergency Medicine, S. Antonio Abate General Hospital, Tolmezzo, Italy

## Abstract

**Introduction:**

The clinical picture of the pandemic influenza A (H1N1)v ranges from a self-limiting afebrile infection to a rapidly progressive pneumonia. Prompt diagnosis and well-timed treatment are recommended. Chest radiography (CRx) often fails to detect the early interstitial stage. The aim of this study was to evaluate the role of bedside chest ultrasonography (US) in the early management of the 2009 influenza A (H1N1)v infection.

**Methods:**

98 patients who arrived in the Emergency Department complaining of influenza-like symptoms were enrolled in the study. Patients not displaying symptoms of acute respiratory distress were discharged without further investigations. Among patients with clinical suggestion of a community-acquired pneumonia, cases encountering other diagnoses or comorbidities were excluded from the study. Clinical history, laboratory tests, CRx, and computed tomography (CT) scan, if indicated, contributed to define the diagnosis of pneumonia in the remaining patients. Chest US was performed by an emergency physician, looking for presence of interstitial syndrome, alveolar consolidation, pleural line abnormalities, and pleural effusion, in 34 patients with a final diagnosis of pneumonia, in 16 having normal initial CRx, and in 33 without pneumonia, as controls.

**Results:**

Chest US was carried out without discomfort in all subjects, requiring a relatively short time (9 minutes; range, 7 to 13 minutes). An abnormal US pattern was detected in 32 of 34 patients with pneumonia (94.1%). A prevalent US pattern of interstitial syndrome was depicted in 15 of 16 patients with normal initial CRx, of whom 10 (62.5%) had a final diagnosis of viral (H1N1) pneumonia. Patients with pneumonia and abnormal initial CRx, of whom only four had a final diagnosis of viral (H1N1) pneumonia (22.2%; *P *< 0.05), mainly displayed an US pattern of alveolar consolidation. Finally, a positive US pattern of interstitial syndrome was found in five of 33 controls (15.1%). False negatives were found in two (5.9%) of 34 cases, and false positives, in five (15.1%) of 33 cases, with sensitivity of 94.1%, specificity of 84.8%, positive predictive value of 86.5%, and negative predictive value of 93.3%.

**Conclusions:**

Bedside chest US represents an effective tool for diagnosing pneumonia in the Emergency Department. It can accurately provide early-stage detection of patients with (H1N1)v pneumonia having an initial normal CRx. Its routine integration into their clinical management is proposed.

## Introduction

The new pandemic influenza A (H1N1) virus emerged in Mexico in April 2009 and has since spread worldwide. The clinical spectrum of presentation ranges from a self-limiting afebrile upper respiratory tract infection to a rapidly progressive lower respiratory tract disease, resulting in intensive care unit (ICU) admission in 25% of patients and in death in 7% [1]. Although underlying comorbidities are common, severe illness has been reported from the 2009 pandemic (H1N1)v infection among young healthy people, including pregnant women [2] and children [3]. Early diagnosis and the consequent start of antiviral treatment is useful in hospitalized patients in reducing disease severity and mortality [1,4].

Pathologic specimens of the initial phases of this disease report an infiltrative interstitial pattern [5], which is not always visible on chest radiography (CRx) [6]. CT scan is considered the gold standard, but its use is limited by radiation exposure, costs, and its frequent unavailability in the emergency setting [7]. The diagnostic use of ultrasound is widely employed in the Emergency Department (ED), thus becoming a standard tool in critical care, strongly recommended by International Societies because it provides a noninvasive, reliable, and low-cost examination [8].

The aim of this study was to evaluate the diagnostic accuracy of chest US for interstitial lung disease and its role in the depiction of early signs of interstitial pneumonia due to the 2009 pandemic influenza A (H1N1)v infection. Indeed, diffuse interstitial lung involvement, although with normal auscultation and CRx, may cause hypoxemia and rapidly generate respiratory failure [9].

## Materials and methods

### Setting and study design

**Figure 1:**
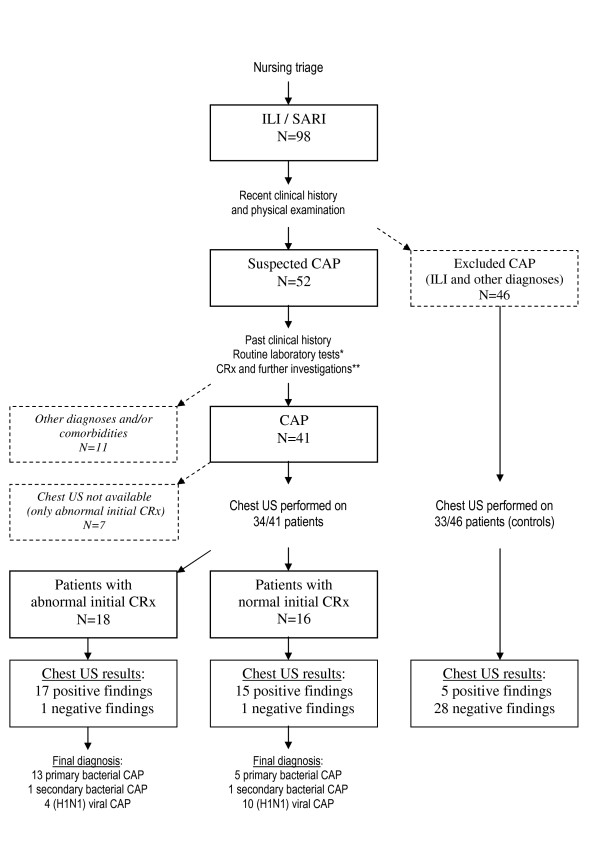
**Study flow-chart**. * Routine laboratory tests included white-cell count and chemical analysis (see text). ** Further investigations included H1N1 test, arterial blood analysis and electrocardiogram; in admitted patients diagnostic specimens from lower respiratory tract and blood cultures were recorded; CT scan and repeated chest radiography, if indicated, were also performed. ILI=influenza like illness; SARI=severe acute respiratory illness; CAP=community-acquired pneumonia; CRx=chest radiography; CT=computed tomography; US=ultrasonography.

This study was conducted in the EDs of A. Gemelli University Hospital (Rome, Italy), Castelnuovo Garfagnana Hospital (Lucca, Italy), and S. Antonio Abate General Hospital (Tolmezzo, Italy). From November 1 to November 30, 2009, we identified 98 consecutive patients (14 years old or older) with suspected 2009 pandemic (H1N1)v infection, complaining of an influenza-like illness (ILI) or severe acute respiratory illness (SARI) at nursing triage, according to WHO guidance [10]. ILI includes sudden onset of fever (> 38°C), cough and sore throat, and rhinorrhea in the absence of other diagnosis. SARI meets ILI case definition and shortness of breath or difficulty breathing, for which hospital admission should be required (Figure 1). Patients complaining of only a clinical picture of ILI, without any symptom or sign of acute respiratory distress, were discharged, not receiving further investigations.

Community-acquired pneumonia (CAP) was clinically suspected on the basis of a longer period of symptoms, presence of cough, fever > 38°C or < 35°C, heart rate > 90 beats per minute, tachypnea > 20 per minute, or dyspnea, abnormal breath sounds as rales and crackles, or abnormal oximetry [11]. Detailed clinical history, routine laboratory tests (complete blood count and differential, glucose, serum electrolytes, liver- and renal-function tests), CRx, and CT scan, if indicated, contributed to confirm the diagnosis of CAP. Patients with other diagnoses or comorbidities potentially affecting chest imaging were excluded from the study [12]. Digital CRx images were obtained in a single posteroanterior view on upright patients (except two cases with Alzheimer disease who had only bedside portable CRx) and independently interpreted by a radiologist and an emergency physician, blind to chest US findings, with the determining support of a third physician, in case of conflicting results.

The 6-point CURB_65 _scale was calculated to assess the severity of CAP [13]. Further investigations included repeated CRx, chest CT scan, diagnostic specimen from the lower respiratory tract, and blood cultures when required. CRx was repeated if the clinical course justified a radiologic investigation to detect worsening of the illness or complications, but was not repeated before discharge in those with a satisfactory clinical recovery from pneumonia. Pretreatment samples of blood, sputum, and urine for microbiologic testing and urine antigen detection were collected from hospitalized patients with severe CAP and clinical indications [12]. Arterial blood gas analysis was obtained in the ED in patients with oximetry < 92%. Laboratory confirmation of 2009 (H1N1)v infection was performed within 0 to 2 days after admission in all CAP patients with a real-time reverse-transcriptase polymerase chain reaction (RT-PCR) assay and viral culture of nasopharyngeal specimens [14].

The final diagnosis of CAP was based on the clinical course of the disease, response to therapy, routine and specific laboratory tests, initial and repeated CRx and CT imaging, when available, not including chest US results, by two independent emergency physicians, supported by a third physician in case of conflicting decisions.

According to standardized criteria [11,15-17], three diagnostic categories were identified: Viral (H1N1) pneumonia in the presence of laboratory confirmation of 2009 influenza (H1N1)v infection; and secondary or primary bacterial pneumonia in the presence of a clinical picture of bacterial infection, with or without laboratory confirmation of viral (H1N1) infection, respectively [17]. Empiric antibiotic and/or specific antiviral treatment was immediately started in all CAP patients, according to their risk stratification, in agreement with international guidelines and recent recommendations [4,15,17]. Chest US was carried out almost simultaneous with CRx (time lag, ≤2 hours). An emergency physician (AT, GS, RC) with more than 10 years of experience in emergency US performed chest US examination, blind to radiologic results, in each ED participating in the study.

### Chest US

#### Ultrasonographic technique

A Toshiba SSA-250A (Tokyo, Japan), an Esaote MyLab 30 (Florence, Italy), and an Esaote Megas CVX (Florence, Italy) ultrasound machine, each equipped with a 3- to 6-MHz convex array transducer, were used. All patients undergoing bedside US scanning were systematically studied in a standardized way and in each lung zone, with longitudinal and transversal scanning. They were examined at the back in a seated position, and anterolaterally, in a supine or semirecumbent position; in two patients in whom the seated position was not possible, a lateral decubitus position was used to examine posterior lung regions. Each hemithorax was divided into five areas: two anterior, two lateral, and one posterior, as previously described [18]. The time for executing each US study was measured.

#### Ultrasonographic appearances

Chest US examination was performed to look for four signs as follows: (1) presence, distribution, and extent of interstitial syndrome; (2) pleural line abnormalities; (3) alveolar consolidation; and (4) pleural effusion.

Interstitial syndrome is characterized by the presence of more than three well-defined B-lines, or by a "white lung" appearance if B-lines are confluent for each examined area. B-lines constitute an US sign of subpleural interlobular septal thickening and are produced by repeated reflection between interfaces of tissues with a large acoustic impedance difference, such as fluid and air. The B-lines increase in thickness and number is strictly related to the entity of extravascular (interstitial) lung water [19]. Pleural-line abnormalities were defined by the thickness of pleural line greater than 2 mm or its coarse appearance, eventually associated with abolished lung sliding, explained by inflammatory adherences due to exudates. Alveolar consolidation is composed of small superficial hypoechoic areas of varying shape with irregular borders, corresponding to fluid-filled alveoli, or large hypoechoic areas (hepatization), often with depiction of air bronchograms, due to massive exudative parenchymal consolidation; disappearance of the pleural line may occur [7,20]. Pleural effusion is defined as anechoic dependent collection limited by diaphragm and pleural layers [18,20].

### Statistics

The study was planned as an observational prospective multicenter trial, with patients' informed consent and approval by hospital ethical committee. The values are presented as median and range (min-max values). Estimates of specificity, sensitivity, and overall accuracy were calculated on subjects submitted to chest US, who constituted the study group. US results were compared with final diagnosis at discharge, assuming ILI patients as controls to calculate "true negative" and "false positive" results, and patients with final CAP diagnosis to calculate "true positive" and "false negative" results. Group differences were analysed by using the χ^2 ^test and the Student *t *test for unpaired data, where appropriate. The test was considered statistically significant if *P *< 0.05. The statistical tests were obtained with computed conventional techniques.

## Results

### Clinical characteristics and outcome measures

A flow diagram of patient selection is reported in Figure 1. CAP was clinically excluded in 46 of 98 patients, who were discharged without further investigations: 33 of them were randomly submitted to chest US, as controls. In 52 of 98 patients, a CAP was suspected, 11 of whom with other diagnoses or comorbidities were excluded from the study. In the remaining 41 patients, a final diagnosis of CAP was confirmed: chest US was carried out in 34 of them, 16 with normal and 18 with abnormal initial CRx findings.

**Figure 2:**
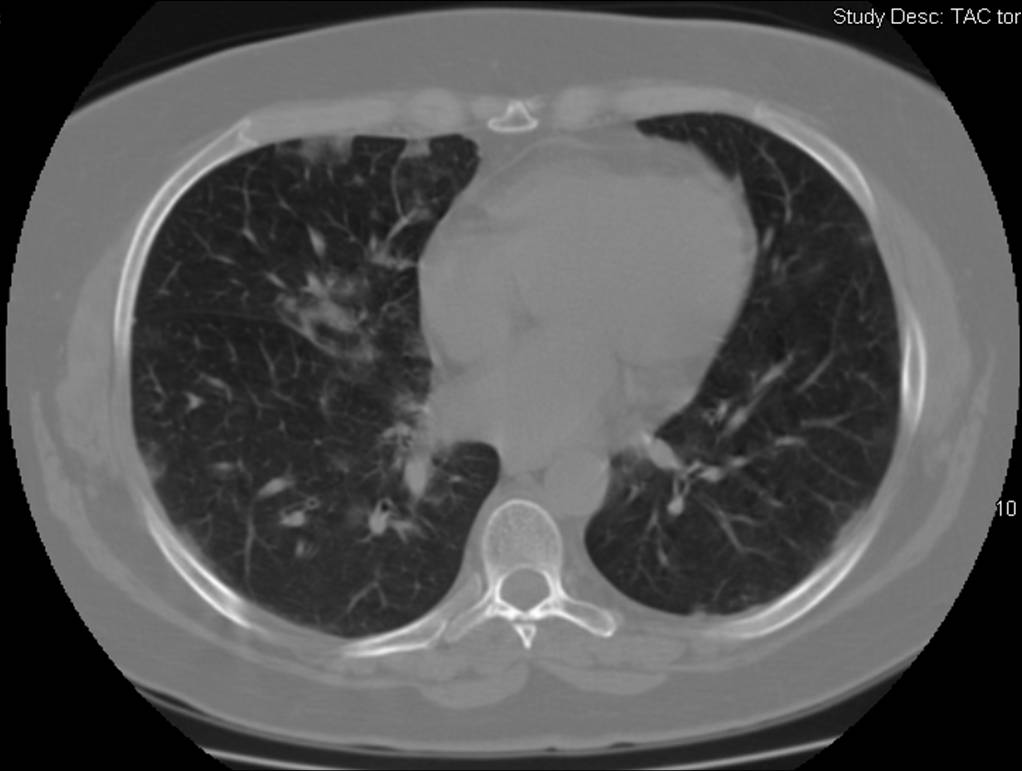
**Chest CT scan shows ill-defined ground-glass opacities with thickened interlobular septa and some peripheral and central ill-defined nodules prevalent at the base in the right lung and diffusely in left lung**.

Main radiologic and US findings and outcome measures, other than baseline clinical characteristics of 16 CAP patients having initial normal CRx (seven women and nine men; median age, 49 years; range, 19 to 85 years), are shown in Table 1. Ten (62.5%) had a final diagnosis of viral (H1N1) CAP. The median length of illness at first evaluation was 3 days (range, 1 to 15 days). Seriated follow-up CRx was available in seven of 16 patients who showed a progression of disease, between the second and fourth days after admission (median, 3 days). Chest CT scans were performed in eight patients at a variable time after their initial CRx, ranging from 0 to 7 days. In four patients, CT scan was available at first evaluation in ED, showing patches of peripheral ground-glass opacities with interlobular septal thickening in all four cases with viral (H1N1) or a secondary bacterial pneumonia diagnosis (bilateral involvement in all cases) (Figure 2). A predominant pattern of parenchymal consolidation was found in the other four patients, with bilateral involvement in two cases. The severity assessment based on the CURB_65 _score resulted in 1 (range, 1 to 3), without a significant difference in ICU-admitted patients.

**Table 1:** Baseline characteristics, imaging results, and outcome measures of patients having CAP diagnosis with initial normal CRx.

Pts	Sex, Age	Chronic illness	Clinical features					Chest US	CT^§ ^(H1N1) LOS/ICU^			Final diagnosis
								
	M/F, yrs		Onset*	Rales	SaO_2_^#^	T(°C)	CURB_65_			test	days/ICU	
Case 1	M, 60	Diabetes	2 days	No	90%	38.2	2	Neg	Pos	+	21/ICU	VP
Case 2	F, 50	Hypertens. Asthma	10 days	No	93%	39.0	1	IS		+	7	VP
Case 3	F, 55	-	3 days	No	96%	38.5	1	IS, PLA	Pos	+	8	SBP
Case 4	F, 31	Hypothiroid.	1 days	Yes	99%	38.0	1	IS, PLA		+	-	VP
Case 5	F, 48	-	3 days	Yes	95%	39.1	1	IS, PLA		+	16	VP
Case 6	F, 85	Hypertens. Alzheimer	15 days	Yes	93%	36.7	3	IS, AC		-	15	PBP
Case 7	M, 34	-	5 days	No	88%	38.4	1	IS, PLA	Pos	+	20	VP
Case 8	M, 73	Hypertens.	4 days	Yes	90%	38.0	3	IS, PE	Pos	-	40/ICU	PBP
Case 9	M, 30	-	3 days	No	88%	38.5	1	IS, PE	Pos	-	10/ICU	PBP
Case 10	M, 44	-	6 days	No	90%	38.2	1	IS, AC, PE	Pos	-	4/ICU	PBP
Case 11	M, 34	-	3 days	No	87%	39.0	1	IS, PLA, AC, PE	Pos	-	18/ICU	PBP
Case 12	F, 60	-	3 days	No	94%	38.5	1	IS, PLA, AC		+	6	VP
Case 13	M, 80	COPD	3 days	No	88%	39.0	3	IS	Pos	+	10	VP
Case 14	M, 37	-	2 days	Yes	95%	39.2	1	IS		+	4	VP
Case 15	F, 62	-	2 days	No	92%	38.7	2	IS, PLA, AC		+	7	VP
Case 16	M, 19	-	3 days	No	94%	38.8	1	IS		+	3	VP

CAP, community-acquired pneumonia; CRx, chest radiography; AC, alveolar consolidation; COPD, chronic obstructive pulmonary disease; IS, interstitial syndrome; PLAs, pleural-line abnormalities; PBP, primary bacterial pneumonia; PE, pleural effusion; SBP, secondary bacterial pneumonia; T, body external temperature; VP, viral pneumonia. ^a^Onset of symptoms before admission to the Emergency Department; ^b^CT scan showed the prevalent pattern of peripheral patch areas of ground-glass opacities; ^c^LOS/ICU, complete hospital stay length and intensive care unit (ICU) admission; ^d^SaO_2_, initial arterial oxygen saturation on room air.

Among 18 CAP patients with an abnormal initial CRx (eight women/10 men; median age, 61 years; range, 14 to 95 years), four had a final diagnosis of viral (H1N1) CAP (22.2%; *P *< 0.05). No significant differences in sex and age resulted between patients with normal and abnormal initial CRx findings. They complained of flu symptoms from 8.5 days (range, 1 to 16 days), significantly longer than did patients with initial normal CRx (*P *< 0.05). Nine among 18 of these patients were treated as outpatients, according to the international recommendations and local guidelines for nonsevere CAP, whereas the remaining nine cases were treated in hospital [4]. The CURB_65 _score (median, 2; range, 0 to 3) did not significantly differ compared with patients with initial normal CRx.

### Chest US findings

The chest US examination was carried out without discomfort in all 67 subjects. It was feasible and required a relatively short time (9 minutes; range, 7 to 13 minutes).

An abnormal US pattern was detected and in 32 (94.1%) of 34 CAP patients, of whom 15 (93.7%) of 16 had normal initial CRx, and in five (15.1%) of 33 ILI patients.

**Figure 3:**
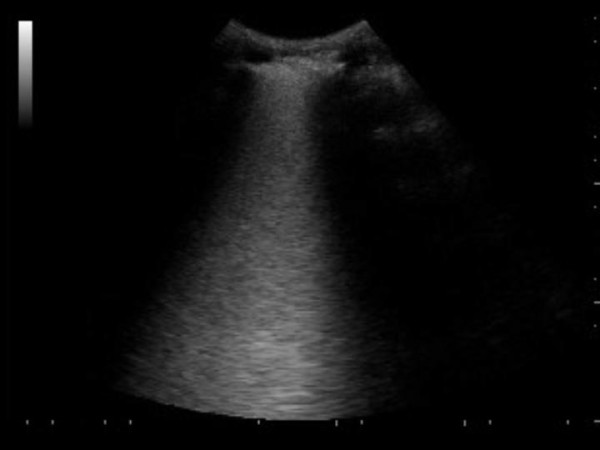
**US pattern displaying well distinct multiple B-lines on anterior chest wall longitudinal scan, defining the interstitial syndrome, is shown. Pleural line thickening is evident**.

**Figure 4:**
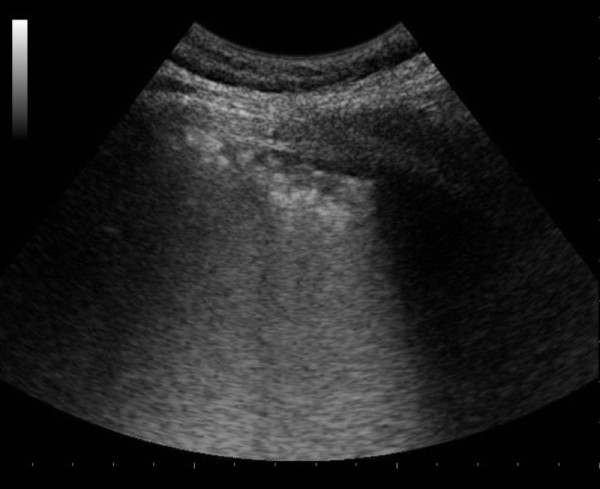
**US pattern displaying confluent B-lines (“white lung”) on lateral middle chest wall scanned longitudinally, coexisting with pleural line thickening, is shown**.

An US interstitial syndrome was found in 10 cases with initial abnormal CRx, of whom eight had viral (H1N1) or secondary bacterial pneumonia and two had primary bacterial pneumonia; alveolar consolidations appeared in the other cases. In some areas, the B-lines were distinct and several (Additional file 1); in others, B lines were run together, producing the US appearance of a "white lung" (Figures 3 and 4 and Additional file 2). The US abnormalities were prevalent in the posterior and lateral fields, especially in the lower halves, with two or more involved distinct areas in 11 cases [nine cases with viral (H1N1) or secondary bacterial pneumonia], and bilateral involvement in nine cases [eight with viral (H1N1) or secondary bacterial pneumonia]. Pleural-line abnormalities were present in almost half the cases, five with viral (H1N1), one with secondary bacterial pneumonia, and one with primary bacterial pneumonia. Small, dependent free pleural effusions were observed in four cases, all with primary bacterial pneumonia. In a case of viral (H1N1) CAP, chest US failed to detect any abnormality.

A prevalent US pattern of alveolar consolidation was found in 17 (94.4%) of 18 CAP patients with an abnormal initial CRx, frequently associated with pleural effusion, but always displaying an interstitial syndrome surrounding the alveolar lesion. Chest US failed to find any abnormality in a case of primary bacterial CAP showing parahilar radiologic consolidation.

An inhomogeneous interstitial syndrome was observed in all ILI patients with a positive US pattern, who were discharged without developing any respiratory disease during follow-up (one case was lost to follow-up).

### Chest US accuracy

The chest US showed false-negative results in diagnosing any CAP in two (5.9%) of 34 cases and false-positive results in five (15.1%) of 33 cases, showing a sensitivity of 94.1% (32 of 34) and a specificity of 84.8% (28 of 33), with 86.5% positive predictive value (32 of 37) and 93.3% negative predictive value (28 of 30).

## Discussion

Bedside chest US findings obtained by emergency physicians in the initial assessment of 2009 pandemic (H1N1)v infection are presented. To date, the role of chest US in (H1N1)v infection has been validated in a single case report of acute respiratory distress syndrome, to optimize ventilatory support and to monitor recovery of lung function by sequential bedside chest US examinations [21].

Chest US showed high accuracy in recognizing lung abnormalities in patients with a final diagnosis of CAP in our study, independent of their initial CRx findings, according to recent reports conducted on patients who presented to the ED for suspected pneumonia [22], as well as on mechanically ventilated patients managed in the ICU [23].

CAP patients with initial normal CRx had a significantly higher percentage of viral (H1N1) pneumonia compared with CAP patients with initial abnormal CRx, consisting with a prevalent radio-occult interstitial involvement in the first group of patients. Moreover, the shorter length of flu symptoms in patients without than in patients with initial CRx abnormalities, likely corresponded to a less-severe degree or an early stage of the disease in the first group. However, the CURB_65 _score did not differ significantly between the two subsets of CAP patients, showing low accuracy to predict ICU admission, according to a recent report [24].

The false-positive results in our subjects, even if referable to occasional findings of a past interstitial pathology or an unknown underlying illness, could be also related to initial interstitial involvement due to (H1N1)v infection, as based on its epidemiologic dominance in the community at that time, although without clinical relevance and resulting in spontaneous recovery, as confirmed in the patients we were able to follow.

The detection of interstitial syndrome with chest US was proposed in 1997 in various lung diseases [25]. The identification of the B-lines pattern in the diagnosis of interstitial syndrome is considered easy to learn (10 or fewer observations), fast to depict (< 5 minutes), and highly feasible, reproducible, and reliable [22,25,26]. Isolated B-lines may also be seen in healthy subjects, especially at the lung bases, but they should be considered abnormal only when multiple (more than three in the same field) or confluent ("white lung") [18], so showing < 3% false-positive results in anterior and upper lateral areas, but reaching about 21% false-positive results in laterobasal areas [27]. Unfortunately, the US interstitial pattern is not specific, being present in situations as cardiogenic pulmonary edema [19], pneumonia [28], acute respiratory distress syndrome [18], lung contusion [29], and lung fibrosis [30]. Bacterial pneumonia is often associated with pleuritic pain and/or abnormal auscultatory findings, so the US abnormalities can be readily identified by a focused goal-directed US view [20,31]. In interstitial pneumonia, the most frequent pattern in viral etiology, chest US examination must be carried out in each lung zone, to distinguish the interstitial syndrome pattern and the spared areas [32]. This approach requires a longer time, but a mean of < 10 minutes in our study, which was carried out by skilled sonographers.

The small number of cases analyzed represents the main limitation of our study. Moreover, the emergency setting could have induced a less-detailed US examination, so affecting its diagnostic accuracy. The CRx obtained only on a posteroanterior plane can reduce its accuracy [7], but really constitutes the standard radiologic investigation available in the emergency setting [22]. The US-technique limitation, instead, was the difficulty to detect central, supradiaphragmatic, retroscapular, or parahilar lung fields because of physical and anatomic obstacles [7].

## Conclusions

In conclusion, bedside chest US can provide early detection of interstitial involvement in (H1N1)v pneumonia, even when the CRx is normal. Its routine integration into clinical management could allow rapid identification of patients who should start pharmacologic treatment. An US interstitial pattern with spared areas is strongly predictive of viral pneumonia [32], corresponding to CT scan findings in several of our patients, in agreement with literature reports [22,25,33]. Further investigations in a larger population call for confirming our preliminary reports and determining the actual clinical relevance of chest US false-positive results in the management of viral pneumonia.

## Key messages

• Other than traditional pleural effusion, chest ultrasonography has recently emerged as an important tool in detecting pneumothorax, parenchymal consolidation, and interstitial syndrome.

• In our study, bedside chest ultrasonography provided early detection of interstitial involvement in (H1N1)v pneumonia.

• Chest ultrasonography was able to recognize lung abnormalities in a high percentage of patients with pneumonia having a normal standard CRx in the ED.

• An inhomogeneous US interstitial-syndrome pattern with spared areas was strongly predictive of interstitial pneumonia corresponding to CT-scan findings.

• Bedside chest US could be considered part of severity assessment in the management of community-acquired pneumonia in the ED.

## Abbreviations

CAP: community-acquired pneumonia; CRx: chest radiography; CT: computed tomography; ED: Emergency Department; ICU: Intensive Care Unit; ILI: influenza-like illness; SARI: severe acute respiratory illness; US: ultrasound.

## Competing interests

The authors declare that they have no competing interests. Patient consent to publish was obtained.

## Authors' contributions

AT and GS conceived the study and designed the trial. NGS obtained research funding. AT and NGS supervised the conduct of the trial, and RG and GP collected data. GS, AT, and RC undertook recruitment of participating centers and patients, performed chest US, and managed the data, including quality control. RG and GP provided statistical advice on study design and analyzed the data; AT chaired the data-oversight committee. RG drafted the manuscript with the kind help of S. Sher (see Acknowledgements). All authors contributed substantially to the revision of the manuscript and read and approved its final version. AT takes responsibility for the manuscript as a whole.

## Supplementary Material

Additional file 1 **Clip 1: Interstitial syndrome**. US pattern displaying distinct multiple B-lines moving together to lung sliding on anterior chest wall longitudinal scan, defining the interstitial syndrome. Pleural-line thickening is evident. Click here for file

Additional file 2 **Clip 2: White lung**. US pattern displaying confluent B-lines ("white lung") moving together to lung sliding on lateral middle chest wall scanned longitudinally, coexisting with superficial alveolar consolidation, pleural-line thickening and "sentry"thin pleural fluid collection. Click here for file
